# Factors Affecting the Duration of Surgery in the Management of Condylar Head Fractures

**DOI:** 10.3390/jcm12227172

**Published:** 2023-11-19

**Authors:** Simon Patrik Pienkohs, Axel Meisgeier, Johannes Herrmann, Leontine Graf, Clarissa S. Reichert, Guilherme Trento, Andreas Neff

**Affiliations:** 1 Department of Oral and Maxillofacial Surgery, University of Marburg, D-35043 Marburg, Germany; 2 Statistical Consulting Services, D-35390 Gießen, Germany; 3 Department of Cranio-Maxillofacial Surgery, University Hospital Münster, D-48149 Münster, Germany

**Keywords:** condylar head fracture, operating time, muscle relaxation, mandibular fracture

## Abstract

Prolonged operation times should be avoided due to the associated complications and negative effects on the efficiency of the use of operating room resources. Surgical treatment of mandibular condylar head fractures is a well-established routine procedure at our department, nevertheless, we recognized fluctuating operating times. This study aims to pinpoint the influencing factors, in particular the hypothesis whether the efficiency of intraoperative muscle relaxation may decisively affect the duration of surgery. It analyses 168 mandibular condylar head fractures that were surgically treated in the period from 2007 to 2022 regarding the duration of the surgery and potential factors affecting it. The potential predictors’ influence on the dependent variable operation time was mainly calculated as a bivariate analysis or linear regression. Efficiency of relaxation (*p* ≤ 0.001), fragmentation type (*p* = 0.031), and fracture age (*p* = 0.003) could be identified as decisive factors affecting the duration of surgery, as the first surgeon was a constant. In conclusion, surgical intervention should start as soon as possible after a traumatic incident. In addition, a dosage regimen to optimize the efficiency of relaxation should be established in future studies. Fragmentation type and concomitant fractures should also be considered for a more accurate estimation of the operating time.

## 1. Introduction

Fractures of the mandibular articular process, including fractures of the condylar head, represent approximately 25 to 30% of all mandibular fractures, therefore constituting the most common mandibular fracture [1,2]. Condylar head fractures themselves account for approx. 10% of all mandibular fractures, or one-third of all fractures of the articular process [1,2,3].

Open reduction and the internal fixation of condylar head fractures is finding increasing favour, irrespective of the location of the fracture, in the event of instability of the vertical dimension—especially in the absence of dental supporting zones—and in dislocated fractures [4,5,6,7,8,9,10,11,12,13]. Especially in the case of multifragmented condylar head fractures, patients can benefit functionally from surgical treatment, especially regarding avoidance of occlusion disorders [5,10,11]; however, surgical treatment of such multifragmented condylar head fractures is routinely performed only at specialized centers because of the considerable effort and expertise required [14].

At the Department of Oral and Maxillofacial Surgery of the University Hospital Giessen and Marburg (UKGM), at its Marburg specialized facility of campus Marburg, surgical care is predominately performed as a standardized procedure [15]. Despite procedure standardization regarding the execution of surgery—including clear assignment of tasks to the staff involved—variability in the duration of the operation is nevertheless marked, which is unappealing from an economic standpoint, as the operating time contributes to the total cost of a surgical procedure [16,17,18,19,20] and has been recognized as a modifiable cost driver in operating room management [21,22,23,24,25,26,27].

Not only is the prolonged duration of surgery undesirable from an economic point of view but it is also associated with an increased risk of intraoperative and postoperative complications [28], such as an increased risk of surgical site infections [29,30,31]. Also, correlations between venous thromboembolism, bleeding, hematoma formation, tissue necrosis, and prolonged duration of surgery have been described [28,32,33,34]. Therefore, due to these adverse consequences for the patient, a reduction in surgery time should be a primary goal of surgical management [28].

Moreover, accurate surgery time prediction is important for efficient operating theatre utilization. Underestimating operating times will lead to extended preoperative waiting times, reduced patient satisfaction, and the necessity to use other hospital-associated resources—such as overtime incurred to be remunerated and the need to provide backup staff—and leading to surgeon burnout [35]. While assessing operating times may be essential for scheduling, it can be difficult to predict, especially in oral and maxillofacial surgery [36]. Accordingly, identifying factors influencing an operation’s duration serves to improve surgery management. 

The aim of this study is to identify factors affecting the duration of the procedures of surgical reduction and osteosynthesis of unilateral and bilateral mandibular condylar head fractures, well-established at our department for many years, in order to be able to explain the variability outlined above in this regard. Based on the results of this study, possible adjustments made with regard to the abovementioned factors may reduce the duration of surgery and may improve predictability in surgery planning in the future. 

In this context, the hypothesis that the efficiency of intraoperative relaxation plays a decisive role in the duration of the operation should be examined in particular. The proximal (viz. “small”) fragment in condylar head fractures is supported by the lateral pterygoid muscle and is usually dislocated anteriorly and medially [37]. The repositioning of this fragment can only be carried out under adequate relaxation [14,37,38]. 

## 2. Materials and Methods

Initial pre-selection of the patient sample investigated was performed using search masks (surgery and procedure codes) and then defined according to inclusion and exclusion criteria. Included were all patients who underwent open reduction and osteosynthesis of a unilateral or bilateral mandibular condylar head fracture at the Department of Oral and Maxillofacial Surgery of the University Hospital of Marburg, a specialized center for temporomandibular joint disorders [15], between 2007 and 2022, performed by the same surgeon (experienced specialist with profound experience in the traumatology of condylar head fractures, senior author A.N.).

Excluded were all patients who received other specialist care due to multiple traumas outside the oral and maxillofacial area during the course of the operation in the same procedure or for whom operation time was not documented.

In order to control for possible distortion of the evaluations due to possibly lengthy surgical treatment of concomitant fractures in the same surgical procedure, the same statistical analysis was again carried out on a sub-sample (N_sub_ = 75).

The inclusion factors of the sub-sample are identical to the complete sample, except that only unilateral mandibular condylar head fractures were considered here.

Thus, in addition, all patients in the sub-sample who received simultaneous surgical treatment of a concomitant fracture in the oral and maxillofacial region in the course of the same surgery or who presented a bilateral condylar head fracture were excluded.

The procedure of open reduction and internal fixation of condylar head fractures at our hands was described previously [15] and can be exemplary and cursorily summarized as accessing the condyle through a retroauricular approach, repositioning the main condylar fragment, sometimes stabilizing and fixating the fragment with microplate osteosynthesis temporarily, and permanently stabilizing the main fragment through screw osteosynthesis, finally recreating the capsule, soft tissue, and external auditory canal. Smaller fragments are fixated with, inter alia, microplates [15,39,40,41,42].

The operation time for this procedure was defined as a reference parameter, as the recorded period from initial skin incision to completion of wound closure.

The following possible influencing factors were determined: patient-related data regarding parameters of age and sex of the patient, fracture age in days, fracture type (non-fragmented, minor fragmented, or major fragmented condylar head fracture) [43,44], the concomitant fractures treated (paramedian, corpus, mandibular angle, condylar base, condylar neck, or simple midfacial fracture), the professional experience of the first surgeon and the first surgical assistant. In addition, the variables describing the experience of the anesthetist, the subjective muscle resistance of the lateral pterygoid muscle during fragment reduction, and the quantity of muscle relaxant (rocuronium) administered were collected in order to assess the degree of influence of muscle relaxation on the duration of surgery.

The morphology of the condylar head fracture was classified by the degree of fragmentation into non-fragmented, minor fragmentation, or major fragmentation, analogous to the AO-CMF trauma classification [43,44].

The variable “experience of the first assistant” was broken down into seven levels according to the years of the assisting resident’s specialist training (training year 1–5 = level 1–5, respectively), if fully trained specialist (level 6), or if senior physician (level 7). 

The variable “experience of anesthetist” was defined as the number of procedures as an attending anesthetist when the procedure was performed (only surgical treatment of unilateral and bilateral mandibular condylar head fractures was counted).

The quantity of muscle relaxant (rocuronium) administered was analyzed as the total amount administered over the entire duration of the operation in relation to the patient’s body weight. A more detailed analysis with regard to the exact point in time of administration, the quantities applied at those specific points in time, or the context of the administration was not possible as reporting standards did not provide for it.

For the subjective muscle resistance of the lateral pterygoid muscle during fragment reduction (subjective assessment of the surgeon according to the surgical report), five levels were defined as very strong resistance, strong, average, weak, and very weak resistance, respectively. The assessment was routinely documented by the first surgeon with regard to the force required to be applied to the instruments for fragment repositioning. 

The statistical analysis was carried out using IBM SPSS Statistics version 26 for Windows. Bivariate analyses were performed between surgery time and potential influencing factors. Due to a significant outlier and the unclear causality between the amount of muscle relaxant administered and the duration of surgery, the relation was calculated using Spearman’s correlation, as was the relationship between muscle resistance and the amount of muscle relaxant administered, and as was the relationship between fracture age and the amount of muscle relaxant administered. The remaining statistical correlations were calculated as mean comparisons or linear regression using the UNIANOVA procedure. In the presence of variance heterogeneity, regressions with robust standard errors (HC3) were requested, and the mean comparisons were analyzed using Welch’s ANOVA with Games–Howell post hoc tests. In cases of variance homogeneity, the analysis was performed using ANOVA, and the pairwise comparisons were adjusted according to Tukey. Mean values with standard deviations are reported for the comparisons of means; unstandardized regression coefficients are reported with a 95% confidence interval for the regressions. In addition, *p*-values and R^2^ (adj.) are reported. R^2^_adj._ serves as a standardized measure of effect strength and can be interpreted according to the suggestions of Cohen (1988)—weak correlation: R^2^_adj._ = 0.01; medium correlation: R^2^_adj._ = 0.06; strong correlation: R^2^_adj._ = 0.14 [45].

## 3. Results

A total of 154 patients with condylar head fractures were treated in the period from 2007 to 2022. Twelve patients had to be excluded from the study as the documentation did not contain surgery time. Another four had to be excluded because they had undergone a parallel surgical procedure allocated to a different medical specialty in the same operation.

Thus, 138 patients were eligible for evaluation.

Not all patient cases could be included in the analysis of certain parameters due to insufficient documentation in the relevant files. Any deviations from the complete collective (N = 138) were considered in the statistical evaluation (cf. Table 1, Table 2, Table 3 and Table 4). 

Thus, the sample comprised 138 patients, of which 72 were male and 66 female (N_sub_: 44 male, 31 female). The mean age was 45.9 years with a standard deviation (SD) of 20.1 and a total age range between 12 and 88 years (N_sub_: M = 43.3 (SD = 19.5), range 14–86 years). The fracture type was predominantly major (n = 80), minor in 48 individuals, and non-fragmented in 40 individuals (N_sub_: 32 major, 24 minor, 19 non-fragmented). 

The 138 patient cases, or 168 condylar head fractures, including unilateral (N = 108) and bilateral (N = 30) condylar head fractures, were evaluated accordingly with regard to surgery time and potential predictors. In addition, an analysis of the sub-sample (N = 75) described above, which only considers unilateral articular head fractures without concomitant fractures treated in the same operation out of the complete collective (N = 138), was also carried out concerning operation time and its possible predictors.

It emerged that the age and the gender of the patient, as well as the experience of the first surgeon, did not statistically affect operating times significantly (cf. Table 1 and Table 2). This was also not the case for the experience of the first assistant or the experience of the anesthetist (cf. Table 1 and Table 2). Concerning the small correlation of R^2^_adj._ = 0.026 in the effect size measurement, it should be noted that it still lies within the random range (*p* = 0.097). 

As to be expected, there was a statistically significant increase in the duration of surgery when concomitant fractures were treated during the same operation. These concomitant fractures included paramedian fractures, corpus, mandibular angle, condylar base, and condylar neck fractures, as well as simple midfacial fractures (cf. Table 2). These presented as individual concomitant fractures or cumulatively alongside the condylar head fracture. Of these, only the fractures treated at the mandibular angle and the condylar base were statistically insignificant, with case numbers of N = 2 and N = 6, respectively (cf. Table 2).

In cases of bilateral mandibular condylar head fractures, the average surgery time (incision to suture) increased from M = 192.1 to M = 338.6 min. In the sub-analysis adjusted for concomitant fractures, the average surgery time was M = 162.3 min (SD 51.4 min for N = 75) per condylar head fracture from initial skin incision to finalization of sutures (cf. Table 2). 

**Figure 1:**
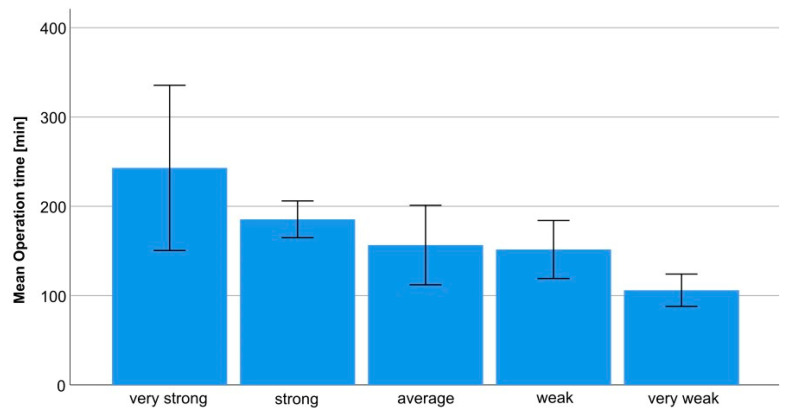
Operation time in relation to different levels of resistance of the lateral pterygoid muscle in the subgroup analysis.

A statistically significant correlation could also be shown between the subjectively assessed muscle resistance of the lateral pterygoid muscle during fragment reduction and the duration of the operation, i.e., an increase or decrease in surgery time by an average of approximately 33 min (all cases) and 27 min (sub-analysis) per negative or positive deviation from the average-value experience (cf. Figure 1).

For each day elapsed without surgical treatment after trauma, surgery time was prolonged by approximately five minutes on average (cf. Table 1). In addition, a statistically relevant correlation emerged between subjective muscle resistance and fracture age in days (cf. Table 3).

**Figure 2:**
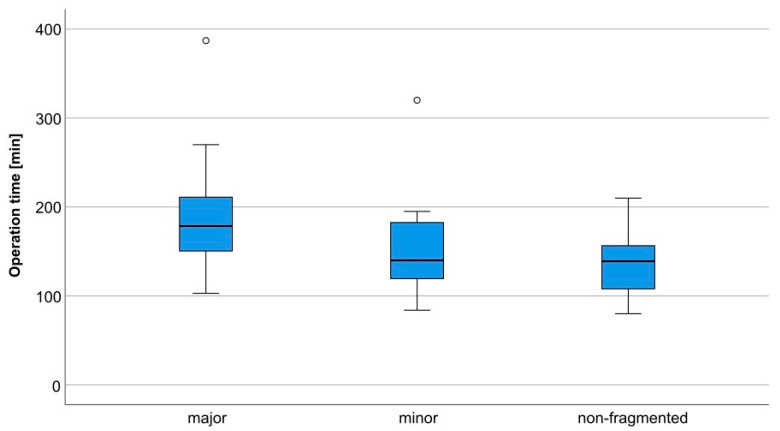
Fragmentation morphology in relation to the operation time in subgroup analysis.

A clear and statistically significant relation between surgery time and fragmentation type could also be established, with surgery time extending with increased severity of fragmentation. In the sub-analysis, the mean surgery time (incision to suture) was 183.8 min for fractures with major fragmentation, 151.2 min for minor fragmentation, and 140.3 min for non-fragmented fractures (cf. Figure 2).

The amount of muscle relaxant (rocuronium) administered also showed a statistically significant correlation (cf. Table 4). It was found that the longer the operation, the more relaxant was administered. Furthermore, there was a significant correlation between the amount of rocuronium administered and fracture age. However, a statistically significant correlation between the amount of muscle relaxant administered and the perceived subjective muscle resistance during fragment reduction could not be proven (cf. Table 4).

## 4. Discussion

The risk of perioperative complications, as reported in the literature, lies between three and seventeen percent for industrialized countries [28,33]. Surgery time has now been identified as an independent and potentially modifiable risk factor and has therefore been the focus of various studies. For example, in 2015, a systematic review by Visser et al. considered prolonged surgery time as one of the three surgery-associated independent risk factors for perioperative complications [46]; however, the results were inconclusive, as out of a mere six studies examined, only three showed a statistically significant association between surgery time and complications [28,46]. In studies in later years, a clearer picture emerged regarding a causal connection between the parameter surgery time and the risk of perioperative complications. Cheng et al. 2018, e.g., describe a redoubled risk of complications when surgery time exceeds two hours and an increase in complications by 14 percent per additional 30 min of surgery time [28]. 

Prolonged surgery time has not only been the focus of attention because of the complications associated with it but also because of its impact on the efficiency of management of operating theatres [17,20,21]. Uncertainties regarding surgery time affect many key performance indicators of the operating theatre and its context [47]. Patient throughput, economic viability, treatment outcome, staff overtime, precise scheduling of subsequent operations, standby times, and patient and practitioner satisfaction are some aspects dependent on the design of surgical scheduling and are affected by an unforeseen extension of surgery duration [47]. 

Realignment of the proximal condylar fragment, which is usually displaced or dislocated anterior-medially in condylar head fractures and supported by the lateral pterygoid muscle, can only be performed under adequate relaxation, as repositioning of this fragment is restricted by muscle traction [14,37,38]. The traction on the reduction instruments, which depends on said relaxation, was documented intraoperatively with the parameter subjective resistance of the lateral pterygoid muscle during fragment reduction. It should be noted here that the first surgeon (A.N.) did the reporting and that the muscle resistance reported was based on his subjective assessment; however, as the first surgeon remained a constant throughout all 168 mandibular condylar head fractures treated, this value was not affected by procedures being performed by different surgeons. Moreover, before his initial condylar head fracture surgery at the UKGM, A.N. had already operated on approximately 200 such condylar head fractures at other institutions [15,48]. This prior experience is displayed statistically, as the experience of the first surgeon was found not to affect surgery time significantly (cf. Table 1). As the study covers a period of 15 years, the possibility of age-related changes to the surgeon cannot be ruled out. Furthermore, the surgeon’s aggregate experience from performing procedures in the operating room over time must also be taken into consideration. In addition, as the muscle resistance was documented by a single surgeon, an intrapersonal bias cannot be ruled out. Nevertheless, the available data of our study suggest a correlation between muscle relaxation and surgery time (cf. Figure 1), confirming our a priori hypothesis.

Also, the quantity of muscle relaxant (rocuronium) administered showed a statistically significant correlation with surgery time. The statistical analysis was conducted here through Spearman’s correlation, as the causal direction was unclear. The result shows that more muscle relaxants were administered during longer operations (cf. Table 3a). However, the documentation provides no answer to the question of whether the bolus administration of muscle relaxant was due to a protracted operation time with difficult reduction under insufficient relaxation or whether the duration of the operation was prolonged despite adequate administration of muscle relaxant. The exact time of administration was not documented in the anesthesiological documentation, and the extent of relaxation in the train-of-four (TOF) was only documented in isolated cases. Neither was the context documented, e.g., whether the muscle relaxant was administered during fragment reduction. Furthermore, no statistically significant correlation emerged between the amount of rocuronium administered and the muscle resistance of the lateral pterygoid muscle during fragment reduction. Thus, further research and specific documentation will be necessary to determine the adequate dosage of muscle relaxant to be applied in the context of fragment reduction.

As expected, the treatment of concomitant fractures in the course of the same operation entailed a significant increase in the duration of the operation, except for fractures of the mandibular angle (N = 2; *p* = 0.156; SD = 213.5 min) and fractures of the condylar base (N = 6; *p* = 0.697; SD = 89.4 min). Concomitant fractures were, however, on occasion also present in combination. The surgery time required to treat such concomitant fractures may exceed the average surgery time for condylar head fractures several times, especially if a multiplicity of fractures presents. For example, the parameter for concomitant treatment of a simple midfacial fracture is stated as M = 427 min, whereas the average surgery time for a simple condylar head fracture is M = 192.1 min (cf. Table 2). Due to the small number of cases of concomitant fractures of a maximum of thirty patients (paramedian fractures) and a minimum of two patients (mandibular angle fractures), in consequence, a sub-analysis was carried out in an attempt to avoid distortions due to surgical treatment of more than one condylar head fracture in one procedure. 

The results of the sub-analysis were no different and did not show any changes from statistically non-significant to significant or vice versa with regard to the analysis of the epidemiological parameters of sex and age. The male-to-female ratio of approximately 52.2% to 47.8% in the full analysis was similar to the gender distribution of the sub-analysis, with 60% male and 40% female patients (cf. Table 2). This is largely consistent with the existing literature, which reports a 2:1 ratio of male to female patients in condylar head fractures [49]. The sex ratio reported for mandibular fractures ranges between 55.8% and 79% for males and 44.2% to 21% for females [50]. The mean age also remained largely identical to the sub-analysis at M = 45.9 years (Msub = 43.3 years), and therefore, above the average age described in the literature of approximately 31 years for condylar head fractures and 33 years for mandibular fractures [49,50].

It should be noted, however, that changes can be observed with regard to the average surgery times. Especially, the lower average surgery time of unilateral condylar head fractures of M = 162.3 min should be emphasized here. In the complete data set analysis, the figure is M = 192.1 min. The reduction by 29.8 min is thus most likely attributable to the treatment of concomitant fractures included in the complete analysis. 

**Figure 3:**
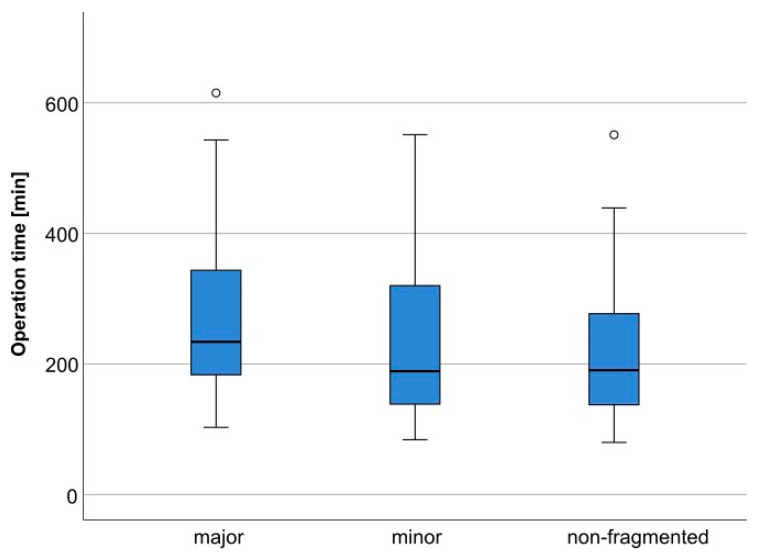
Fragmentation morphology in relation to the operation time in complete sample analysis.

The most striking difference between the sub-analysis and analysis of the complete data set emerged concerning the different types of fragmentation. Here, the complete data set analysis shows no significance in the relation between major and minor fragmentation. The sub-analysis, however, shows a statistically significant result with a *p*-value of 0.039 (cf. Table 2). This is probably due to the fact that the total operation time of a bilateral condylar head fracture is attributed to the fractures treated regardless of the degree of fragmentation, as the individual operation time per side is not documented, which may lead to distortions in resulting values. Therefore, especially regarding the type of fragmentation, the focus should be on the sub-analysis that excluded bilateral condylar head fractures. Nevertheless, the degree of fragmentation generally correlates significantly with operation time, and a relevant difference is also evident in the descriptive results. Treatment of a major fragmented fracture compared to a minor fragmented fracture requires an additional 38 min on average (sub-analysis 32.6 min), compared to a non-fragmented fracture which requires as much as 52.2 min (sub-analysis 43.5 min) (cf. Table 2 and Figure 2 and Figure 3).

The proportion of major fragmented fractures of 47.6% in the collective studied deviates significantly from the probability of 10–15%, as reported in the literature [14]. This can most likely be attributed to the fact that the treatment of multifragmented articular head fractures is usually only routinely carried out at specialized centers [14]. Therefore, supraregional referrals are frequently made to our specialized center for the surgical treatment of multifragmented condylar head fractures to ensure that the aims of osteosynthesis and the best possible restoration of the undisturbed anatomical and functional pre-trauma situation can succeed, despite the considerable effort and the special expertise required. In this respect, the operating times determined in our study must be evaluated, taking on board the aspect that fractures with a high degree of fragmentation are treated without exemption and were not excluded from surgical treatment as in other studies [9,51,52].

Given our results, referral and surgical treatment of affected patients should occur promptly to avoid increased surgical time, which increases by approximately 5 min with each day that elapses after the initial trauma (cf. Table 1). This can best be explained by physiological remodeling processes that occur after a traumatic injury to the muscle. Cells involved in the healing process of the muscle, including inflammatory cells and stem cells, may degrade matrix proteins by emitting matrix metalloproteases and elastase [53,54,55,56]. The onset of this process is the immediate day of the trauma [53]. As early as one week post-trauma, in the course of the healing process, scar formation begins in the musculature by fibroblasts in response to local mediators such as TGF-β1 [53,57]. Therefore, the statistically significant correlation between fracture age and subjective muscle resistance during fragment reduction is unsurprising (cf. Table 3).

Thus, operating time reduction can be achieved by performing surgical treatment of mandibular condylar head fractures as quickly as possible. Fracture age and degree of fragmentation should be included as predictors of operating time in preoperative planning in order to achieve a more precise estimate of operating time and thus, probably, a more efficient use of the operating theatre and an improved reliability of planning. Optimized muscle relaxation during fragment reduction is a promising approach to achieve a significant reduction in the operating time with the associated benefit of avoiding complications. Research into the ideal dosage regimen and, if necessary, the choice of specific muscle relaxant should therefore continue. 

A limitation of our study is its limited significance, as already described above, with regard to the amount of muscle relaxant (rocuronium) to be administered in order to achieve adequate muscle relaxation, as documentation of the exact time of administration in the anesthesiological reports was insufficient and the context of the administration of relaxants can not be established. Further studies are required to understand the causality between the amount of muscle relaxant and the duration of surgery and to determine the appropriate amount of relaxant to optimize the procedure. 

Another limitation of this study is that it does not offer a comparative analysis of the effectiveness of different muscle relaxants in the context of reduction and osteosynthesis of unilateral and bilateral mandibular condylar head fractures, as only rocuronium could be referred to in our study.

Also, in every instance of surgical treatment of the 168 mandibular condylar head fractures examined in this study, a retroauricular approach was used exclusively. A statement about operating times for the predominantly used preauricular approach can therefore not be made and would need to include functional outcome parameters since the operation’s success depends decisively on careful soft tissue management [14]. It cannot be ruled out that surgery time for the retroauricular approach, with its clear advantages over the preauricular approach and notably better soft tissue protection, differs from surgery time for the latter as such [5,58]. 

Moreover, a possible bias cannot be ruled out as patients were excluded when no operation time was documented or they received other specialist care due to multiple traumas outside the oral and maxillofacial area during the course of the same operation. Especially for the latter type of operation, as the different procedures performed were not documented individually regarding their duration, an investigation regarding the severity of overall trauma in correlation to operating time could not be executed. 

Furthermore, as this was a single-center study, the sample size of the study is limited to the 168 cases of condylar head fractures described, or 75 fractures in the sub-sample, and has not been complemented with cases from other centers.

In addition to that, our study does not present figures specifying the cost of the operative time and, accordingly, could not display the reduction or increase in accumulated costs for decreased or increased operating time, respectively. In future studies, a cost-efficiency analysis could provide a way to evaluate the economic gain through reduced operative times.

## 5. Conclusions

Based on the results of our study, efficiency of relaxation, fragmentation type, and fracture age are decisive factors for the duration of surgery when the first surgeon remains a constant. A dosage regimen to optimize the administration of relaxants must be discussed in further studies. Concomitant fractures treated in the same operation and treatment of two condylar head fractures in one single surgery will extend the duration of the operation accordingly and should be factored in during surgery planning. 

## Figures and Tables

**Table 1:** Linear associations (regression), dependent variable: surgery time (min).

	Complete Sample (N = 138)				Subgroup Analysis Unilateral CHF (N = 75)			
Predictor	Valid N	b (CI)	*p*	R^2^adj.	Valid N	b (CI)	*p*	R^2^adj.
Patient age	138	0.21 (−0.67–1.10)	0.637	−0.006	75	−0.41 (−1.02–0.20)	0.186	0.010
Fracture age (d)	132	5.1 (1.8–8.3)	0.003	0.061	74	3.4 (1.0–5.8)	0.006	0.088
Surgeon experience (y)	138	1.98 (−2.33–6.29)	0.366 *	−0.002	75	−0.87 (−3.51–1.76)	0.512 *	−0.009
Assistant experience (y)	129	−4.6 (−15.7–6.5)	0.413	−0.003	70	−4.9 (−10.7–0.9)	0.097	0.026
Anesthetist experience (c)	130	−0.68 (−2.46–1.10)	0.450	−0.004	71	−0.17 (−1.33–0.99)	0.771	−0.013
Muscle resistance	137	−33.0 (−47.6–−18.5)	<0.001 *	0.124	74	−27.1 (−36.5–−17.7)	<0.001	0.303

b: unstandardized regression coefficient. CI: 95% confidence interval for b. * robust standard errors (HC3). (c): count, number of performed operations.

**Table 2:** Mean comparisons, dependent variable: duration of surgery (min).

		Complete Sample (N = 138)				Subgroup Analysis without Concomitant Fractures (N = 75)			
Predictor		Valid N	M (SD)	*p*	R^2^adj.	Valid N	M (SD)	*p*	R^2^adj.
Sex	m	72	213.4 (99.4)	0.220	0.004	44	162.3 (53.5)	0.988	−0.014
	f	66	235.4 (110.9)			31	162.5 (49.1)		
Bilateral fracture	no	108	192.1 (83.2)	<0.001	0.328				
	yes	30	338.6 (96.6)						
Fracture type	major	80	267.7 (114.8)	0.031 *	0.030	32	183.8 (53.8)	0.005 **	0.114
	minor	48	229.7 (110.8)			24	151.2 (48.2)		
	non-fragmented	40	215.5 (101.9)			19	140.3 (37.4)		
Concomitant fracture treated: paramedian	no	108	203.1 (94.3)	<0.001	0.136				
	yes	30	298.9 (109.7)						
Concomitant fracture treated: corpus fracture	no	131	218.4 (101.0)	0.007	0.045				
	yes	7	326.9 (136.3)						
Concomitant fracture treated: mandibular angle fracture	No	136	222.4 (103.6)	0.156	0.008				
	Yes	2	329.0 (213.5)						
Concomitant fracture treated: fracture of condylar base	No	132	223.2 (106.1)	0.697	−0.006				
	Yes	6	240.3 (89.4)						
Concomitant fracture treated: condylar neck fracture	No	118	214.7 (101.0)	0.012	0.039				
	Yes	20	278.4 (116.1)						
Concomitant fracture treated: simple midfacial fracture	No	133	216.3 (95.5)	<0.001	0.135				
	Yes	5	427.0 (157.0)						

M: mean. SD: standard deviation. * ANOVA; pairwise comparisons, Tukey: major-minor: *p* = 0.147; major-simple: *p* = 0.042; minor-simple: *p* = 0.822. ** ANOVA; pairwise comparisons, Tukey: major-minor: *p* = 0.039 major-simple: *p* = 0.007; minor-simple: *p* = 0.742.

**Table 3:** Correlation (Spearman’s) between the age of the fracture and resistance of the lateral pterygoid muscle.

	Complete Sample (N = 138)			Subgroup Analysis without Concomitant Fractures (N = 75)		
	Fracture Age (d)			Fracture Age (d)		
	rho	*p*	Valid N	rho	*p*	Valid N
Muscle resistance	−0.340	<0.001	131	−0.233	0.047	73

**Table 4:** Correlation (Spearman’s) between the amount of rocuronium administered, the operation time, the age of the fracture, and the resistance of the lateral pterygoid muscle.

	Complete Sample (N = 138)			Subgroup Analysis without Concomitant Fractures (N = 75)		
	Amount of Rocuronium Administered			Amount of Rocuronium Administered		
	rho	*p*	Valid N	rho	*p*	Valid N
Duration of surgery (min)	0.335	<0.001	129	0.3304	0.005	71
Muscle resistance	−0.140	0.114	128	−0.212	0.078	70
Fracture age (d)	0.317	<0.001	129	0.265	0.025	71

## Data Availability

The data presented in this study are available on request from the corresponding author.

## References

[1] Hlawitschka M., Eckelt U. (2002). Assessment of patients treated for intracapsular fractures of the mandibular condyle by closed technics. J. Oral Maxillofac. Surg..

[2] Rasse M. (2000). Neuere Entwicklungen der Therapie der Gelenkfortsatzbrüche der Mandibula. Mund Kiefer Gesichtschir..

[3] Kozakiewicz M., Walczyk A. (2023). Current Frequency of Mandibular Condylar Process Fractures. J. Clin. Med..

[4] He D., Yang C., Chen M., Jiang B., Wang B. (2009). Intracapsular condylar fracture of the mandible: Our classification and open treatment experience. J. Oral Maxillofac. Surg..

[5] Kolk A., Neff A. (2015). Long-term results of ORIF of condylar head fractures of the mandible: A prospective 5-year follow-up study of small-fragment positional-screw osteosynthesis (SFPSO). J. Craniomaxillofac. Surg..

[6] McLeod N.M., Saeed N.R., Gerber B. (2023). Remodelling of mandibular condylar head after fixation of fractures with ultrasound activated resorbable pins: A retrospective case series. J. Craniomaxillofac. Surg..

[7] Hirjak D., Machon V., Beno M., Galis B., Kupcova I. (2017). Surgical treatment of condylar head fractures, the way to minimize the postraumatic TMJ ankylosis. Bratisl. Lek. Listy..

[8] Kozakiewicz M. (2018). Small-diameter compression screws completely embedded in bone for rigid internal fixation of the condylar head of the mandible. Br. J. Oral Maxillofac. Surg..

[9] Xie S.-T., Singhal D., Chen C.-T., Chen Y.-R. (2013). Functional and radiologic outcome of open reduction and internal fixation of condylar head and neck fractures using miniplate or microplate system. Ann. Plast. Surg..

[10] Yao S., Zhou J., Li Z. (2014). Contrast analysis of open reduction and internal fixation and non-surgical treatment of condylar fracture: A meta-analysis. J. Craniofac. Surg..

[11] Eckelt U., Schneider M., Erasmus F., Gerlach K.L., Kuhlisch E., Loukota R., Rasse M., Schubert J., Terheyden H. (2006). Open versus closed treatment of fractures of the mandibular condylar process—A prospective randomized multi-centre study. J. Craniomaxillofac. Surg..

[12] Chrcanovic B.R. (2012). Open versus closed reduction: Diacapitular fractures of the mandibular condyle. Oral Maxillofac. Surg..

[13] Rikhotso R.E., Reyneke J.P., Nel M. (2022). Does Open Reduction and Internal Fixation Yield Better Outcomes Over Closed Reduction of Mandibular Condylar Fractures?. J. Oral Maxillofac. Surg..

[14] Neff A. (2019). Open reduction and internal fixation in temporomandibular joint traumatology: Current concepts and future perspectives. Stomatol. Dis. Sci..

[15] Neff A., Kolk A., Meschke F., Deppe H., Horch H.H. (2005). Kleinfragmentschrauben vs. Plattenosteosynthese bei Gelenkwalzenfrakturen. Vergleich funktioneller Ergebnisse mit MRT und Achsiographie [Small fragment screws vs. plate osteosynthesis in condylar head fractures]. Mund Kiefer Gesichtschir..

[16] Bronheim R.S., Shu H.T., Jami M., Hsu N.N., Aiyer A.A. (2023). Surgical Setting in Achilles Tendon Repair: How Does It Relate to Costs and Complications?. Foot Ankle Orthop..

[17] Patel S., Lindenberg M., Rovers M.M., van Harten W.H., Ruers T.J.M., Poot L., Retel V.P., Grutters J.P.C. (2022). Understanding the Costs of Surgery: A Bottom-Up Cost Analysis of Both a Hybrid Operating Room and Conventional Operating Room. Int. J. Health Policy Manag..

[18] Ayloo S.M., Buchs N.C., Addeo P., Bianco F.M., Giulianotti P.C. (2011). Traditional versus single-site placement of adjustable gastric banding: A comparative study and cost analysis. Obes. Surg..

[19] Hagen M.E., Pugin F., Chassot G., Huber O., Buchs N., Iranmanesh P., Morel P. (2012). Reducing cost of surgery by avoiding complications: The model of robotic Roux-en-Y gastric bypass. Obes. Surg..

[20] Doble B., Wordsworth S., Rogers C.A., Welbourn R., Byrne J., Blazeby J.M., By-Band-Sleeve Trial Management Group (2017). What Are the Real Procedural Costs of Bariatric Surgery? A Systematic Literature Review of Published Cost Analyses. Obes. Surg..

[21] Garras D.N., Beredjiklian P.K., Leinberry C.F. (2011). Operating on a stretcher: A cost analysis. J. Hand Surg. Am..

[22] Rastogi S., Paul S., Kukreja S., Aggarwal K., Choudhury R., Bhugra A., Indra B N.P., Jawaid M. (2017). Treatment of Mandibular Angle Fractures with Single Three-Dimensional Locking Miniplates without Maxillomandibular Fixation: How Much Fixation Is Required?. Craniomaxillofac. Trauma Reconstr..

[23] Mittal Y., Varghese K.G., Mohan S., Jayakumar N., Chhag S. (2016). A Comparative Study of 3-Dimensional Titanium Versus 2-Dimensional Titanium Miniplates for Open Reduction and Fixation of Mandibular Parasymphysis Fracture. J. Maxillofac. Oral Surg..

[24] Bokshan S.L., Mehta S., DeFroda S.F., Owens B.D. (2019). What Are the Primary Cost Drivers of Anterior Cruciate Ligament Reconstruction in the United States? A Cost-Minimization Analysis of 14,713 Patients. Arthroscopy.

[25] Proietti L., Ciolli G., Corona K., Cerciello S. (2021). Regarding “What Are the Primary Cost Drivers of Anterior Cruciate Ligament Reconstruction in the United States? A Cost-Minimization Analysis of 14,713 Patients”. Arthroscopy.

[26] Deviandri R., van der Veen H.C., Lubis A.M.T., Utoyo G.A., van den Akker-Scheek I., Postma M.J. (2022). Burden and Cost of Anterior Cruciate Ligament Reconstruction and Reimbursement of Its Treatment in a Developing Country: An Observational Study in Indonesia. Clinicoecon Outcomes Res..

[27] Huyke-Hernández F.A., Siljander B., Flagstad I., Only A., Parikh H.R., Tompkins M., Nelson B., Kweon C., Cunningham B. (2022). Cost and Cost Driver Analysis of Anterior Cruciate Ligament Reconstruction Using Time-Driven Activity-Based Costing: Bone-Tendon-Bone Autograft Versus Hamstring Autograft. JBJS Open Access.

[28] Cheng H., Clymer J.W., Po-Han Chen B., Sadeghirad B., Ferko N.C., Cameron C.G., Hinoul P. (2018). Prolonged operative duration is associated with complications: A systematic review and meta-analysis. J. Surg. Res..

[29] Cheng H., Chen B.P., Soleas I.M., Ferko N.C., Cameron C.G., Hinoul P. (2017). Prolonged Operative Duration Increases Risk of Surgical Site Infections: A Systematic Review. Surg. Infect..

[30] Leong G., Wilson J., Charlett A. (2006). Duration of operation as a risk factor for surgical site infection: Comparison of English and US data. J. Hosp. Infect..

[31] Schoenfeld A.J., Carey P.A., Cleveland A.W., Bader J.O., Bono C.M. (2013). Patient factors, comorbidities, and surgical characteristics that increase mortality and complication risk after spinal arthrodesis: A prognostic study based on 5,887 patients. Spine J..

[32] de’Angelis N., Schena C.A., Piccoli M., Casoni Pattacini G., Pecchini F., Winter D.C., O’Connell L., Carcoforo P., Urbani A., Aisoni F. (2022). Impact of operation duration on postoperative outcomes of minimally-invasive right colectomy. Color. Dis..

[33] Shah N., Hamilton M. (2013). Clinical review: Can we predict which patients are at risk of complications following surgery?. Crit. Care..

[34] Tranchart H., Gaillard M., Chirica M., Ferretti S., Perlemuter G., Naveau S., Dagher I. (2015). Multivariate analysis of risk factors for postoperative complications after laparoscopic liver resection. Surg. Endosc..

[35] Katt B.M., Tawfik A., Lau V., Padua F., Fletcher D., Stamos B., Aita D., Conte E., Saxena A., Hornstein J. (2021). The Planning Fallacy in the Orthopedic Operating Room. Cureus.

[36] Laskin D.M., Abubaker A.O., Strauss R.A. (2013). Accuracy of predicting the duration of a surgical operation. J. Oral Maxillofac. Surg..

[37] Rasse M. (1993). Diakapituläre Frakturen der Mandibula. Eine neue Operationsmethode und erste Ergebnisse. Z. Stomatol..

[38] Rasse M. (1992). Diakapituläre Frakturen der Mandibula. Die Operative Versorgung—Tierexperiment und Klinik.

[39] Axhausen G. (1931). Die operative Freilegung des Kiefergelenks. Chirurg.

[40] Bockenheimer P. (1920). Eine neue Methode zur Freilegung der Kiefergelenke ohne sichtbare Narben und ohne Verletzung des Nervus facialis. Zentralbl Chir..

[41] Kermer C., Undt G., Rasse M. (1998). Surgical reduction and fixation of intracapsular condylar fractures: A follow up study. Int. J. Oral Maxillofac. Surg..

[42] Umstadt H.E., Ellers M., Müller H.-H., Austermann K.H. (2000). Functional reconstruction of the TM joint in cases of severely displaced fractures and fracture dislocation. J. Craniomaxillofac. Surg..

[43] Loukota R.A., Neff A., Rasse M. (2010). Nomenclature/classification of fractures of the mandibular condylar head. Br. J. Oral Maxillofac. Surg..

[44] Neff A., Cornelius C.P., Rasse M., Audigé L. (2017). Kiefergelenkfortsatzfrakturen nach der AO-CMF-Trauma-Klassifikation. Die MKG-Chir..

[45] Cohen J. (1988). Statistical Power Analysis for the Behavioral Sciences.

[46] Visser A., Geboers B., Gouma D.J., Goslings J.C., Ubbink D.T. (2015). Predictors of surgical complications: A systematic review. Surgery.

[47] Kayis E., Wang H., Patel M., Gonzalez T., Jain S., Ramamurthi R.J., Santos C., Singhal S., Suermondt J., Sylvester K. (2012). Improving prediction of surgery duration using operational and temporal factors. AMIA Annu. Symp. Proc..

[48] Neff A., Kolk A., Deppe H., Horch H.H. (1999). Neue Aspekte zur Indikation der operativen Versorgung intraartikulärer und hoher Kiefergelenkluxationsfrakturen [New aspects for indications of surgical management of intra-articular and high temporomandibular dislocation fractures]. Mund Kiefer Gesichtschir..

[49] Marker P., Nielsen A., Bastian H.L. (2000). Fractures of the mandibular condyle. Part 2: Results of treatment of 348 patients. Br. J. Oral Maxillofac. Surg..

[50] Depprich R., Handschel J., Hornung J., Meyer U., Kübler N.R. (2007). Causation, therapy and complications of treating mandibular fractures—A retrospective analysis of 10 years. Mund Kiefer Gesichtschir..

[51] Smolka W., Cornelius C.P., Lechler C. (2018). Resorption behaviour of the articular surface dome and functional outcome after open reduction and internal fixation of mandibular condylar head fractures using small-fragment positional screws. J. Craniomaxillofac. Surg..

[52] Yang W.G., Chen C.T., Tsay P.K., Chen Y.R. (2002). Functional results of unilateral mandibular condylar process fractures after open and closed treatment. J. Trauma.

[53] Forcina L., Cosentino M., Musarò A. (2020). Mechanisms Regulating Muscle Regeneration: Insights into the Interrelated and Time-Dependent Phases of Tissue Healing. Cells.

[54] Davis M.E., Gumucio J.P., Sugg K.B., Bedi A., Mendias C.L. (2013). MMP inhibition as a potential method to augment the healing of skeletal muscle and tendon extracellular matrix. J. Appl. Physiol..

[55] Kim J., Lee J. (2016). Matrix metalloproteinase and tissue inhibitor of metalloproteinase responses to muscle damage after eccentric exercise. J. Exerc. Rehabil..

[56] Arecco N., Clarke C.J., Jones F.K., Simpson D.M., Mason D., Beynon R.J., Pisconti A. (2016). Elastase levels and activity are increased in dystrophic muscle and impair myoblast cell survival, proliferation and differentiation. Sci. Rep..

[57] Garg K., Corona B.T., Walters T.J. (2015). Therapeutic strategies for preventing skeletal muscle fibrosis after injury. Front. Pharmacol..

[58] Neff A., Neff F., Kolk A., Horch H.H. (2001). Conference Papers-Evaluation of risks and treatment complications in open TMJ-surgery. Dtsch. Zahnärztliche Z..

